# Toxicity of Gossypol from Cottonseed Cake to Sheep Ovarian Follicles

**DOI:** 10.1371/journal.pone.0143708

**Published:** 2015-11-24

**Authors:** Antônio Carlos Lopes Câmara, Ivana Cristina Nunes Gadelha, Pedro Augusto Cordeiro Borges, Silvano Alves de Paiva, Marília Martins Melo, Benito Soto-Blanco

**Affiliations:** 1 Hospital Veterinário, Universidade Federal Rural do Semi-Árido (UFERSA), Av. Francisco Mota 572, Mossoró, RN, 59625-900, Brazil; 2 Departamento de Clínica e Cirurgia Veterinárias, Escola de Veterinária, Universidade Federal de Minas Gerais (UFMG), Avenida Antônio Carlos 6627, Belo Horizonte, MG, 30123-970, Brazil; Alexandria University, EGYPT

## Abstract

Gossypol, a polyphenol compound produced by cotton plant, has proven reproductive toxicity, but the effects of gossypol on sheep ovaries are unknown. This study was aimed to determine the *in vitro* and *in vivo* effects of gossypol on the ovarian follicles of sheep. This trial was divided into two experiments. In the first one, we used twelve non-pregnant, nulliparous, Santa Inês crossbred ewes, which were randomly distributed into two equal groups and fed diets with and without cottonseed cake. Feed was offered at 1.5% of the animal’s body weight for 63 days. The concentrations of total and free gossypol in the cottonseed cake were 3.28 mg/g and 0.11 mg/g, respectively. Throughout the trial period, no animal showed clinical signs of toxicity and no effects on body weight were observed. However, there was a significantly lower number of viable ovarian follicles (20.6%) and higher number of atretic follicles (79.4%) in the gossypol-fed sheep compared to the control (85.1 and 34.9%, respectively). These findings were observed at all stages of follicular development. In the second experiment, eight ovaries from slaughterhouse were cultured with different concentrations of gossypol acetic acid (0, 5, 10 and 20 μg/mL) for 24 hours or seven days. The *in vitro* action of gossypol resulted in a significant decrease in viable ovarian follicles, especially the primary and transition follicles, and a significant increase in the number of atretic follicles after 24 hours of culture. These follicles were greatly affected when cultured with gossypol for seven days. It is concluded that gossypol present in cotton seeds directly acts on ovarian follicles in sheep to increase atresia.

## Introduction

Gossypol (2,2-bi(8-formyl-1,6,7-trihydroxy-5-isopropyl-3-methylnaphthalene)) is a phenolic compound produced by the pigment glands of cotton (*Gossypium* spp.) and is found mostly in the seeds of the plant. Ruminant rations frequently include cotton seed cake and meal resulting in exposure to gossypol. Unfortunately, however, gossypol is known for its toxic effects including reproductive toxicity [1,2].

Several studies have demonstrated that gossypol has deleterious effects on spermatogenesis by inhibiting sperm motility and decreasing spermatozoa concentration in semen, inducing mitochondrial lesions in spermatozoa flagella and causing germinal epithelial damage [2–8]. These effects depend on the dose and length of exposure, with reversion of these effects in bulls occurring 7 months after cessation of gossypol ingestion [9]. The mechanism underlying these effects in spermatozoa also includes the blockage of the production, release and utilization of ATP [10]. Furthermore, gossypol exposure inhibits the influx of calcium and the activities of the Mg-ATPase and Ca-Mg-ATPase enzymes on the plasma cell membranes of spermatozoa [11].

Gossypol interferes with the estrus cycle of rodents [12–14] and impairs the steroid synthesis by granulosa cells in pigs [15]. In addition, gossypol interrupted early pregnancy and initial embryonic development in cattle [16–20]. In rats, it was verified that gossypol induces the degeneration of ovarian follicles, decreasing the proportion of viable follicles and increasing atretic follicles at all stages of follicular development [14]. However, whether these effects occur in ruminants has not been determined. This study was aimed to determine the effects of gossypol on the ovarian follicles of sheep that were fed cottonseed cake. Furthermore, sheep ovaries were cultured with gossypol to determine whether these effects are directly promoted by the compound.

## Materials and Methods

### Ethics statement

Animal studies were approved by the Institutional Animal Care and Use Committee at the Universidade Federal Rural do Semi-Árido/UFERSA (process 23091.003174/2014-61) and were performed in accordance with Brazilian regulations for the care and use of laboratory animals.

### Experiment 1

#### Animals and experimental design

The experiment was conducted at the Mossoró municipality, Rio Grande do Norte state, Northeastern Brazil (05°11'16"S and 37°20'38"W), from September to November 2014.

We used 12 clinically healthy, non-pregnant nulliparous crossbred Santa Inês ewes that were 1 to 3 years old and weighed 28.3 ± 4.90 kg. Before starting the experiment, sheep were submitted to two deworming procedures at a 21-day interval (5 mg/kg of Albendazol, Biozen^®^, Biofarm, Jaboticabal, SP, Brazil) and were kept in the experimental conditions for 30 days. During the entire study period, the animals were housed in pens of 3.0 x 3.0 m^2^ (two sheep per pen). Tifton (*Cynodon dactylon*) hay, mineral salt and tap water were provided *ad libitum*.

Two rations formulated to contain 18% crude protein were used as feed. The control ration was composed of 750 g/kg of corn and 250 g/kg of soybean meal, and the treatment ration contained 510 g/kg of corn, 90 g/kg of soybean meal and 400 g/kg of cottonseed cake. The rations were offered at 1.5% of the body weight and were provided twice daily over 63 consecutive days. Animals were randomly assigned to two different treatments: animals fed with the treatment ration (n = 6; treated group) and animals fed with the control ration (n = 6; control group). The concentrations of free and bound gossypol in the cottonseed cake were determined using the UV-HPLC method [21,22].

After 63 days of treatment, all sheep were subjected to a bilateral ovariectomy according to the methodology described by Rodrigues and colleagues [23] with some modifications. The ewes were submitted to intramuscular dissociative anesthesia (xylazine: 0.1 mg/kg and ketamine: 2.0 mg/kg), placed on dorsal recumbence and routinely prepared for aseptic surgery. Infiltrative anesthesia with 10 mL of lidocaine was made in the alba line approximately 5–10 cm above the udder. Then a 8–10 cm incision was done and the ovaries and uterus were pulled with an Allis clamp. Hemostasis was accomplished by the use of 200 x 4.8 mm nylon cable ties on the ovarian pedicles. Afterward the linea alba was sutured with Vycril 2–0 in a double continuous pattern and the skin was sutured with Nylon 0 and separated Wolf pattern. The ewes were submitted to anti-inflammatory (flunixin meglumine: 2.2 mg/kg, intramuscularly, once a day for 3 days) and antibiotic (enrofloxacin: 5 mg/kg, intramuscularly, once a day for 5 days) therapy. The surgical wounds were cleaned daily with physiologic solution and sprayed with topical repellent and healing powder (Bactrovet^®^, König). Skin sutures were removed 14 days after surgery. The ovaries were individually weighed and measured and then fixed in Carnoy solution for 12 hours for a subsequent histological analysis and staining with hematoxylin and eosin (H&E).

#### Morphological analysis of the ovarian follicles from control and gossypol-fed sheep

The ovaries from all sheep were collected at the end of the first experiment and were fixed in Carnoy solution for 12 hours. Five-micron thick paraffin-embedded sections were collected at 60 μm intervals throughout the tissue and stained with H&E. A histological analysis was performed, including qualitative and quantitative assessments of the ovarian follicles. Follicles were classified according to their stage of development as primordial, primary, secondary or antral [24]. Primordial follicles presented one layer of flattened granulosa cells, transition follicles one layer of both flattened and cuboidal granulosa cells, primary follicles one layer of cuboidal granulosa cells, secondary follicles two layers of cuboidal granulosa cells, and antral follicles an antral cavity.

Follicles were also classified as viable or atretic. The viable follicles presented a regular shape and well-organized granulosa cells, without signs of atresia. The atretic follicles were characterized by the presence of retracted oocytes, pyknotic nuclei, a discontinuous basement membrane, and disorganized granulosa cells [25,26].

### Experiment 2

The experiment was conducted in September 2014. Eight ovaries were obtained from four clinically healthy, 3- to 5-year-old crossbred Santa Inês ewes collected at a slaughterhouse in Mossoró municipality, Rio Grande do Norte state, Brazil. The ovaries were fragmented and cultured in 24-well cell culture plates containing 1 mL of culture medium. One fragment of each ovary was allocated to each treatment. The culture medium consisted of alpha minimum essential medium (α-MEM), Eagle’s medium supplemented with 2 mM glutamine, 2 mM hypoxanthine, 1.25 mg/ml bovine serum albumin BSA, 50 μg/mL ascorbic acid (Sigma, Sigma-Aldrich, St Louis, MO, USA), and antibiotics (100 μg/mL of penicillin and 100 μg/mL of streptomycin, Gibco, Grand Island, NY, USA). Four concentrations of (+/-)-gossypol acetic acid (G4382, Fluka, Buchs, Switzerland) were tested: 0, 5, 10 and 20 μg/mL. The plates were incubated at 39°C in 5% CO_2_ (Panasonic CO_2_ Incubator MCO-18AC, Leicestershire, UK) for either 24 hours or 7 days. The culture medium was replaced every 48 hours. After incubation, the ovaries were fixed and processed for a histological analysis using H&E staining.

Approximately 300 follicles per treatment were counted and classified. The follicles were classified according to the stage of development (primordial, transitional, primary, secondary or antral) and as viable or atretic, following the same classification used in the *in vivo* assay.

### Statistical analyses

The obtained data were statistically analyzed using the R (version 3.1.3) [27] and SAS (version 9.0) software programs. The normality of the data was evaluated using a Shapiro-Wilk test, and the homogeneity of variance was evaluated using an F test. Welch’s t-test was employed for the comparison of dimensions and weight of ovaries, and Mann-Whitney test for comparison of the body weight gain and the number of ovarian follicles. The frequencies of viable and atretic follicles were compared using Fisher’s exact test. Statistical analysis of body weight was carried out using a mixed linear model approach of SAS, using first-order autocorrelation covariate structure. Animals were considered as a random factor, with each animal nested within treatments, with repeated measurements over time. Significant day versus treatment interactions were examined using the PDIFF procedure with preplanned comparisons. *P* values < 0.05 were considered to indicate significance.

## Results

### Experiment 1

The total and free gossypol levels in the cottonseed cake were 3.28 and 0.11 mg/g, respectively. The average dose of consumed free gossypol was 0.66 mg/kg BW/day, whereas the dose of the bound form was 19.7 mg/kg BW/day.

Animals did not show any sign of toxicity during the period of study as evaluated by clinical examinations and by hematological and biochemical analyses (data not shown). Initial and final body weights and body weight gain were 27.3±6.38, 33.4±6.72 and 6.1±3.56 kg in control and 29.4±3.04, 36.9±4.29 and 7.52±1.78 kg in treated sheep. Body weights and body weight gain were not significantly (*p* > 0.05) different.

After ovariectomy, the length (1.00±0.26 cm in control and 0.91±0.24 cm in treated sheep), width (0.61±0.18 cm in control and 0.54±0.16 cm in treated sheep) and weight (1.02±0.38 g in control and 0.82±0.38 g in treated sheep) of ovaries did not show any significant (*p* > 0.05) differences between the groups.

**Fig 1:**
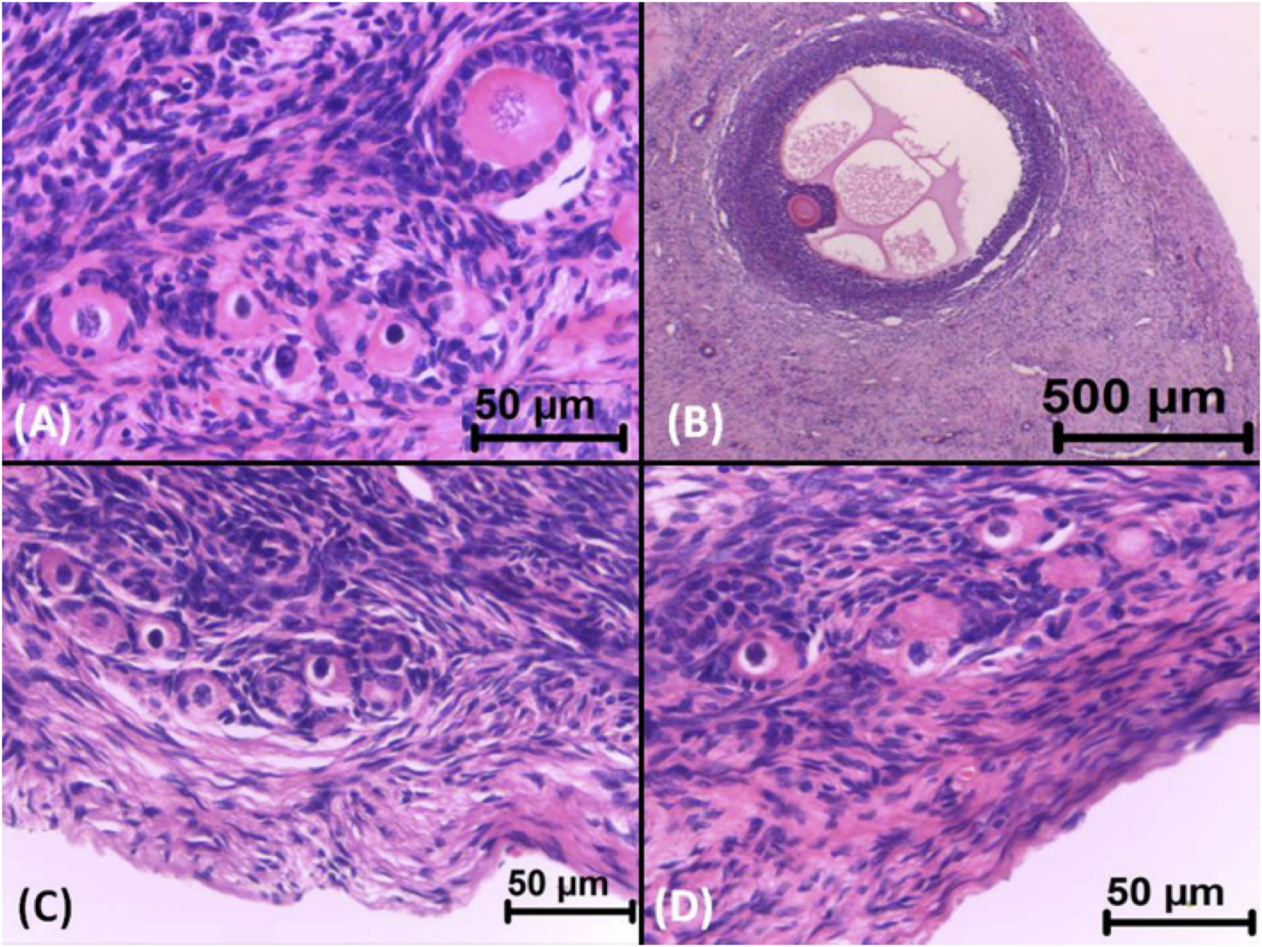
Ovarian follicles of sheep fed rations containing 0 (control group) or 40% (treated group) cottonseed cake. Viable primordial (A—H&E stain, bar = 50 μm) and antral (B—H&E stain, bar = 500 μm) follicles from control sheep. Atretic primordial follicles (C and D) from treated sheep showing retracted oocytes, a pyknotic nucleus, a discontinuous basement membrane and disorganized granulosa cells (H&E stain, bar = 50 μm).

Microscopic evaluation revealed that gossypol-fed animals had a significant (*p* < 0.05) reduction in the number of viable follicles and an increase in atretic follicles at all stages of development (Table 1 and Fig 1).

**Table 1:** Number and proportions of ovarian follicles in sheep fed rations containing 0 (control group) or 40% (treated group; free gossypol: 0,66 mg /kg BW/day) cottonseed cake for 63 days. The data are presented as the means ± standard deviation.

Follicles	Control	Treated
Primordial		
viable	46.8 ± 17.5	12.3 ± 4.80*
	66.7% (281/421)	20.9% (74/354)
atretic	23.3 ± 16.5	46.7 ± 16.5*
	33.3% (140/421)	79.1% (280/354)
Transition		
viable	185.2 ± 35.3	67.5 ± 14.0*
	62.5% (1111/1779)	19.9% (405/2032)
atretic	111.3 ± 16.7	271.2 ± 28.1*
	37.5% (668/1779)	80.1% (1627/2032)
Primary		
viable	32.7 ± 12.2	5.33 ± 3.20*
	76.9% (196/255)	30.5% (32/105)
atretic	9.83 ± 5.60	12.2 ± 6.40
	23.1% (59/255)	69.5% (73/105)
Secondary		
viable	3.33 ± 1.63	0.33 ± 0.52*
	80% (20/25)	13.3% (2/15)
atretic	0.83 ± 1.60	2.17 ± 2.23
	20% (2/25)	86.7% (13/15)
Antral		
viable	4.00 ± 2.90	1.00 ± 0.63*
	85.7% (24/28)	42.9% (6/14)
atretic	0.67 ± 0.52	1.33 ± 1.21
	14.3% (2/28)	57.1% (8/14)
Total		
viable	272.0 ± 32.1	86.5 ± 19.1*
	65.1% (1632/2508)	20.6% (519/2520)
atretic	146.0 ± 27.1	333.5 ± 22.8*
	34.9% (876/2508)	79.4% (2001/2520)

* *p* < 0.05, Mann-Whitney test.

### Experiment 2

**Fig 2:**
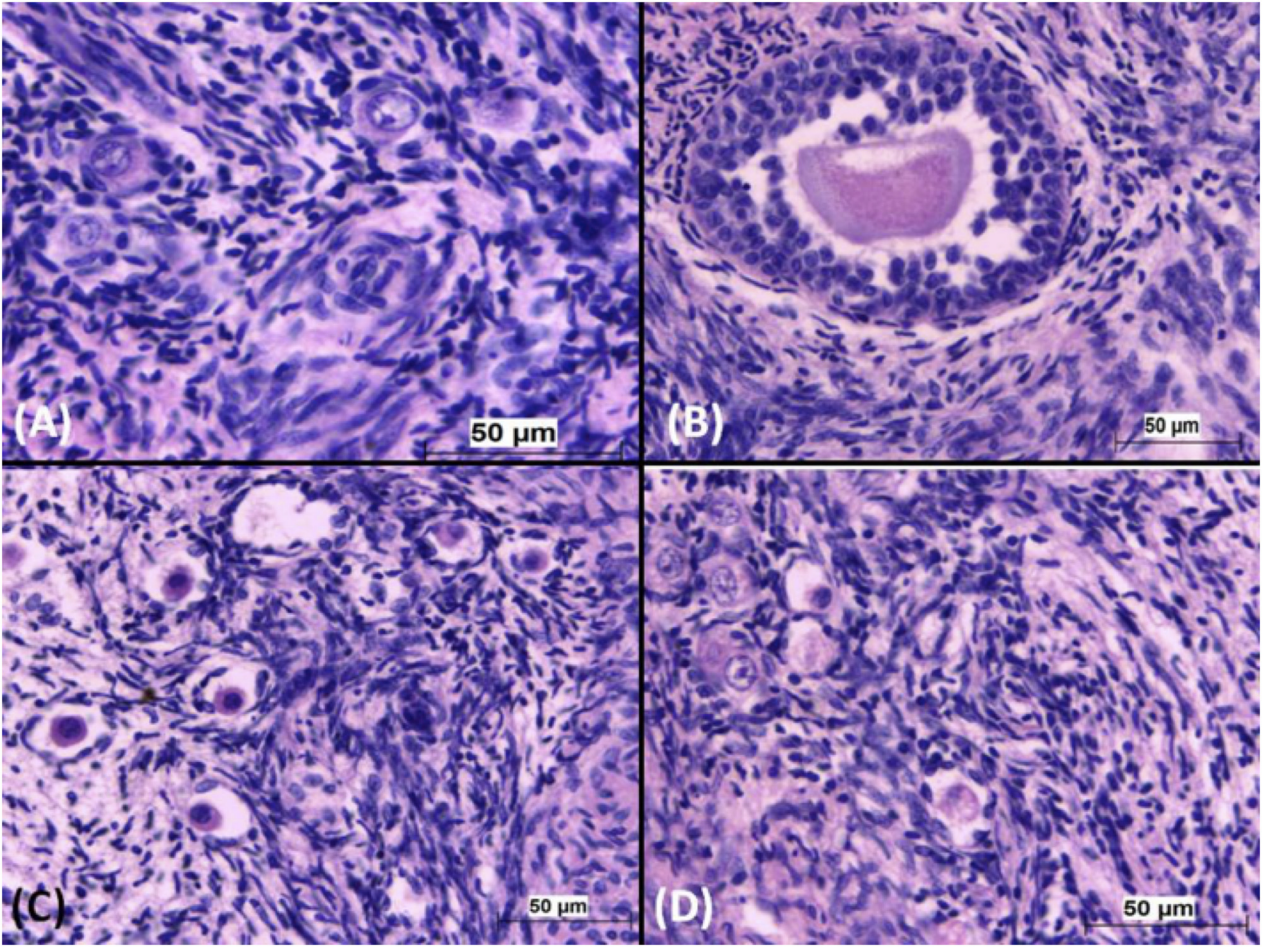
Ovarian follicles from cultured sheep ovaries. Viable primordial (A) and transition (B) follicles from ovaries cultured without gossypol. Atretic primordial follicles (C and D) from ovaries cultured with 10 μg/mL gossypol. H&E stain, bar = 50 μm.

The proportions of the different types of sheep ovarian follicles cultured in the absence (0 μg/mL) and increasing concentrations of gossypol (5, 10 and 20 μg/mL) for 24 hours and seven days are shown in Tables 2 and 3, respectively. Gossypol exposure led to a clear and significant reduction in the number of viable follicles in all stages of development, with a significant increase in the number of atretic follicles (Fig 2) after 24 hours of culture. The same reduction occurred in ovarian follicles cultured with gossypol for seven days, with the exception of antral follicles because there was a limited sample size for the statistical analysis. The frequencies of viable and atretic follicles exposed to gossypol showed significant differences compared to the control treatment, but no significant difference was found between the different concentrations of gossypol for 24 hours and 7 days.

**Table 2:** The populations of ovarian follicles from fragment sheep ovaries cultured with different concentrations of gossypol acetic acid (0, 5, 10, and 20 μg/mL) for 24 hours. Data from approximately 300 follicles per treatment.

Follicles	0 μg/mL	5 μg/mL	10 μg/mL	20 μg/mL	*p* ^a^
Primordial (%)					
viable	67.5 (54/80)	23.2 (22/95)	19.3 (22/114)	17.4 (12/69)	<0.0001
atretic	32.5 (26/80)	76.8 (73/95)*	80.7 (92/114)*	82.6 (57/69)*	
Transition (%)					
viable	71.7 (134/187)	27.2 (46/169)	27.2 (41/151)	23.2 (41/177)	<0.0001
atretic	28.3 (53/187)	72.8 (123/169)*	72.8 (110/151)*	76.8 (136/177)	
Primary (%)					
viable	83.8 (31/37)	13.6 (3/22)	22.6 (7/31)	11.8 (4/34)	<0.0001
atretic	16.2 (6/37)	86.4 (19/22)*	77.4 (24/31)*	88.2 (30/34)*	
Secondary (%)					
viable	75 (3/4)	0	0	0	<0.0001
atretic	25 (1/4)	100 (3/3)	100 (5/5)*	100 (21/21)*	
Antral (%)					
viable	80 (4/5)	16.7 (1/6)	14.3 (1/7)	0	0.005
atretic	20 (1/5)	83.3 (5/6)	85.7 (6/7)	100 (9/9)*	
Total (%)					
viable	72.2 (226/313)	24.4 (72/295)	23.1 (71/308)	18.4 (57/310)	<0.0001
atretic	27.8 (87/313)	75.6 (223/295)*	76.9 (237/308)*	81.6 (253/310)*	

^a^ Fisher’s exact test;

* significant difference (*p* < 0.05) with control (0 μg/mL).

**Table 3:** The populations of ovarian follicles from fragment sheep ovaries cultured with different concentrations of gossypol acetic acid (0, 5, 10, and 20 μg/mL) for seven days. Data from approximately 300 follicles per treatment.

Follicles	0 μg/mL	5 μg/mL	10 μg/mL	20 μg/mL	*p* ^a^
Primordial (%)					
viable	58.1 (18/31)	17.9 (12/67)	24 (18/75)	13 (6/46)	<0.0001
atretic	41.9 (13/31)	82.1 (55/67)*	76 (57/75)*	87 (40/46)*	
Transition (%)					
viable	60.6 (129/213)	10.6 (19/180)	9.9 (19/192)	11.4 (17/149)	<0.0001
atretic	39.4 (84/213)	89.4 (161/180)*	90.9 (173/192)*	88.6 (132/149)*	
Primary (%)					
viable	66.7 (32/48)	10.8 (4/37)	11.5 (3/26)	10.5 (10/95)	<0.0001
atretic	33.3 (16/48)	89.2 (33/37)*	88.5 (23/26)*	89.5 (85/95)*	
Secondary (%)					
viable	87.5 (7/8)	0	0	0	<0.0001
atretic	12.5 (1/8)	100 (11/11)*	100 (4/4)*	100 (10/10)*	
Antral (%)					
viable	66.7 (2/3)	0	0	0	-
atretic	33.3 (1/3)	100 (5/5)	0	100 (1/1)	
Total (%)					
viable	62 (188/303)	11.7 (35/300)	13.5 (40/297)	11 (33/301)	<0.0001
atretic	38 (115/303)	88.3 (265/300)*	86.5 (257/297)*	89 (268/301)*	

^a^ Fisher’s exact test;

* significant difference (*p* < 0.05) with control (0 μg/mL).

## Discussion

In the present study, the average dose of consumed free gossypol was just 0.66 mg/kg BW/day, whereas the dose of the bound form was 19.7 mg/kg BW/day. Although of this free gossypol dose was less than those used in calves and heifers earlier [17,28], bound gossypol might have contributed to the observed toxic effects since that bound forms of gossypol could release free gossypol during digestion [2]. In fact, similar total plasma gossypol levels were observed in cows fed diets containing whole cottonseed with similar total gossypol concentrations but different free gossypol concentrations [29]. The gossypol-fed animals did not exhibit any clinical signs of toxicity or interference with body weight or weight gain. This result is similar to those previously reported in studies of sheep [30,31] and goats [32].

In this study, treatment with cottonseed promoted no significant difference in the dimensions or the weights of the ovaries. However, gossypol was responsible for a significant reduction in the number of viable ovarian follicles and a significant increase in the number of atretic follicles in all stages of development. These data are similar to those found in rats treated with gossypol (25 mg/kg/day subcutaneously), which caused a significant reduction in the total population of ovarian follicles with an increased frequency of atretic follicles [14]. A reduced number of large ovarian follicles (larger than 5 mm) was observed in heifers that received 5 g of free gossypol/animal/day [17]. On the other hand, heifers treated with approximately 51 mg of free gossypol/kg BW/day did not show interference with their cycling, first service conception rate, or ovarian morphology [28]. Thus, it is feasible to speculate that ewes are more sensitive to gossypol than cows. It is not possible now to affirm whether follicular damage is reversible and if it affects fertility, which deserves to be the subject of future studies.

In this study, the *in vitro* assays confirmed the *in vivo* observations that gossypol promotes atresia of ovarian follicle cells. In addition, there was an absence of viable antral follicles after culturing in the presence of gossypol after only seven days. Therefore, it is feasible that exposure to gossypol could result in a drastic reduction in the production of oocytes by sheep ovaries. The proportion of viable primordial follicles was less than half of that observed in the control group. These results are concerning because the number of primordial ovarian follicles in female mammals is fixed at birth. The primordial follicles develop into primary, secondary and antral follicles in a constant and irreversible process. Damage to primary, secondary and antral follicles can lead to temporary infertility when primordial follicles are not affected. Damage to the primordial follicles can result in permanent infertility due to the possible depletion of the pool of these follicles [33]. Therefore, it is possible that exposure to gossypol can reduce the reserve of ovarian follicles and negatively affect female fertility [14]. In addition, our results show that the deleterious effects of gossypol on ovarian follicles occur through the direct action of the compound and not through any product of biotransformation.

The mechanism by which gossypol promotes the damage to follicles is not yet known. Several studies have shown that gossypol has cytotoxic activity [3,12]. This cytotoxic effect can be promoted by the generation of reactive oxygen species, leading to oxidative stress [4,34], an interruption of intercellular communication [35], induction of apoptosis [36,37], and interference with the transport of ions through membranes [8,11,38,39]. Other potential mechanisms underlying the toxicity of gossypol include interference with the cellular energetic metabolism [6,10,40]. In addition to the cytotoxic effects, gossypol promotes a reduction in steroidogenesis, decreasing the serum levels of progesterone and estradiol [14,15]. These facts suggest that the deleterious effects of gossypol on ovarian follicles may result from the interaction of cytotoxic and hormonal factors.

## Conclusions

Our data show that the gossypol present in cottonseed cake can reduce the viability and consequently increase the atresia of ovarian follicles in sheep. This effect was observed in animals with no clinical manifestations, changes in weight, or changes in the hematological and biochemical findings; thus, it may be difficult to assess potential reproductive damage in farms. It was hypothesized that the adverse effects of gossypol on the ovarian follicles are caused by the direct actions of the compound.
